# Dangerous Illuminants

**Published:** 1899-02-25

**Authors:** 


### Dangerous Illuminants.

The report of a Departmental Committee of the Home Office upon the manufacture and use of water-gas, and other gases containing a large proportion of carbonic oxide, together with the reply made by Mr. Jesse Collings, M.P., to a deputation from the Federation of Grocers' Associations, on the subject of the storage and use of petroleum, cannot fail to direct public attention to the many dangers of life and health which may be incidental to improved methods of domestic or industrial illumination. These methods have of late years been carried to such a degree of excellence that the artificial light which satisfied our fathers would not for a moment be tolerated by our contemporaries, who expect to be able to read small print, or to pursue fine handicrafts, with as much ease by night as by day; and who seldom realise that the fulfilment of their desires is attainable only at the cost of some risk to human life, or even of some definite sacrifice of it. During the last few years the manufacture of illuminating gas from coal has been largely supplemented by its manufacture from water, the proportion of water-gas in the United States at present amounting to more than 70 per cent. of the entire consumption; while, in Great Britain, it already amounts to as much as 24 per cent., and is steadily increasing. Both forms of gas contain a certain proportion of the deadly carbonic oxide, which, in an approximately pure condition, is used to fill the "lethal chamber" now employed for the speedy and painless destruction of superfluous dogs, and which, if inhaled by human beings, even in a state of considerable dilution, is speedily productive of serious or fatal consequences. In ordinary coal-gas the carbonic oxide ranges from 4 to 12 per cent. of the total volume; but in water-gas this percentage is increased to 30 or even more, and the dangerous character of leakages, or of other sources of escape, is increased in a corresponding ratio. According to figures furnished to the State Legislature of Massachusetts, it appears that, in the city of Boston, in the year 1886, in round numbers, thirteen thousand million cubic feet of coal-gas (no other kind being then in use) were supplied to twenty-nine thousand customers, and no deaths or accidents were recorded. In 1897 twenty-three thousand million cubic feet, containing 93 per cent. of water-gas, were supplied to eighty thousand customers, and there were forty-five deaths, of which thirty-one were accidental and fourteen were suicidal. The combustion of water-gas is as free from danger as that of coal-gas, and, as regards inanimate objects, books, pictures, furniture, and the like, is probably less injurious by reason of the absence of sulphur; but leakages are dangerous almost in proportion to the amount of carbonic oxide contained, and, as the pure gas is inodorous, they do not reveal themselves when this is used, as it may be, for various heating and manufacturing purposes. Fortunately this pure gas is scarcely an illuminant, and in order to become so it must be "carburetted," or charged with vapourised oil, from which it acquires a strong and characteristic odour. The Departmental Committee report strongly in favour of legislation calculated to diminish the dangers attendant upon the use of water-gas by providing that it shall not be mixed with coal-gas in excess of a definite percentage, and that it shall not be supplied for any purposes unless it has been so treated as to possess a distinct and pungent smell. They also recommend that power should be given to local authorities to exercise control over gas fittings, and to see, especially in lodging-houses and manufactories, that they are of such a kind as to secure as far as may be the safety of inmates and workers.

The question with which Mr. Jesse Collings had to deal was of a more familiar kind; inasmuch as what are called "lamp accidents" have now for many years been reported with great frequency. The right honourable gentleman was able to say that the report of the Select Committee on the subject was receiving the careful consideration of the Government; and he hinted, not very obscurely, that one result of that consideration would probably be to deprive England of her unique position as the only civilised country in which there are no regulations with regard to the sale or storage of petroleum oil. It would, probably, be still more important to have a legislative prohibition of the sale of unsafe lamps. The conditions of safety are well known to manufacturers, and, at the cost of a very few more pence for each lamp, could easily be secured to the purchaser. It is manifest that the whole subject of artificial illumination, in view of the extensive changes and practical improvements of late years, calls imperatively for supervision and control.

